# Using inertial measurement units to estimate spine joint kinematics and kinetics during walking and running

**DOI:** 10.1038/s41598-023-50652-w

**Published:** 2024-01-02

**Authors:** Benjamin E. Sibson, Jacob J. Banks, Ali Yawar, Andrew K. Yegian, Dennis E. Anderson, Daniel E. Lieberman

**Affiliations:** 1 Department of Human Evolutionary Biology, Harvard University, Cambridge, MA USA; 2 Center for Advanced Orthopedic Studies, Beth Israel Deaconess Medical Center, Boston, MA USA; 3 Department of Orthopedic Surgery, Harvard Medical School, Boston, MA USA

**Keywords:** Musculoskeletal system, Biomedical engineering

## Abstract

Optical motion capture (OMC) is considered the best available method for measuring spine kinematics, yet inertial measurement units (IMU) have the potential to collect data outside the laboratory. When combined with musculoskeletal modeling, IMU technology may be used to estimate spinal loads in real-world settings. To date, IMUs have not been validated for estimates of spinal movement and loading during both walking and running. Using OpenSim Thoracolumbar Spine and Ribcage models, we compare IMU and OMC estimates of lumbosacral (L5/S1) and thoracolumbar (T12/L1) joint angles, moments, and reaction forces during gait across six speeds for five participants. For comparisons, time series are ensemble averaged over strides. Comparisons between IMU and OMC ensemble averages have low normalized root mean squared errors (< 0.3 for 81% of comparisons) and high, positive cross-correlations (> 0.5 for 91% of comparisons), suggesting signals are similar in magnitude and trend. As expected, joint moments and reaction forces are higher during running than walking for IMU and OMC. Relative to OMC, IMU overestimates joint moments and underestimates joint reaction forces by 20.9% and 15.7%, respectively. The results suggest using a combination of IMU technology and musculoskeletal modeling is a valid means for estimating spinal movement and loading.

## Introduction

Low back pain (LBP), a multifactorial musculoskeletal (MSK) condition that is the most common cause of years lived with disability, often arises from activities requiring large forces that exceed the tolerances of tissues in the back including muscles, ligaments, and intervertebral discs^1,2^. Activities that moderately stress these tissues have been shown to improve their strength and fatigue-resistance, thereby increasing injury tolerance and reducing the risk of LBP^3,4^. Many studies have tested how different activities load the spine. The most common way to quantify spine loading incorporates inverse dynamics methods that integrate recorded kinetic and kinematic data from ground reaction forces (GRFs) and optical motion capture (OMC) systems^5–8^. Because in vivo spine joint forces are impractical to measure directly, it has also become increasingly common for researchers to develop and validate non-invasive MSK models of the back^9–16^. These models incorporate participant-specific anatomical details, kinematics, and principles of engineering, biomechanics and physics to non-invasively estimate spine tissue loading. One of the most detailed models for the estimation of spine joint kinetics is the full-body Thoracolumbar Spine and Ribcage model, available in the opensource MSK modeling software OpenSim^12,17–22^.

OMC is generally considered the best method for measuring human kinematics. However, OMC systems are expensive and require highly controlled laboratory settings. In real-world contexts, inertial measurement unit (IMU) systems have the potential to record kinematics, but first need to be validated for their usefulness. As with the reflective markers used for OMC, IMUs adhere to a participant’s body and track their segment kinematics; unlike OMC, IMUs can collect kinematic data outside of the laboratory. Several studies have shown IMUs to be a valid alternative to OMC for measuring upper and lower limb kinematics^23–27^. Fewer studies have shown IMUs can provide accurate estimates of three-dimensional (3D) joint kinetics^28,29^. A systematic review on validating IMU-based kinetics reported only 10 of 37 studies that examined the potential of IMUs to investigate spine kinetics^28^. All 10 studies reported good agreement between IMU and OMC estimates of spine joint kinetics. A particularly relevant study compared IMU and OMC estimates of lumbar and thoracic joint moments and forces during walking and found normalized root mean squared error (*NRMSE*) values of 0.06–0.17 (corresponding to errors of 6–17% of the OMC range)^30^. Other previous work focused on manual materials handling tasks or static poses^28^.

IMUs have not been validated for estimates of 3D kinematics, moments, and reaction forces from the thoracolumbar spine using a MSK model during walking and running. Doing so has the potential to improve understanding of LBP etiology. Prospective studies have shown that engaging regularly in moderately intense physical activities such as brisk walking helps prevent LBP^31–33^. A proposed mechanism for this reduced vulnerability to LBP is the development of increased trunk muscle fatigue resistance, a strong predictor of first-time and recurrent LBP that is associated with physical activity (PA)^34–37^. How individuals load their spines may also influence their LBP risk, yet current research on the effects of PA on spine tissue loading has considered only participants from postindustrial environments. Individuals in postindustrial environments are much less likely to engage in evolutionarily normal PAs such as walking long distances, carrying moderate loads like food and water, and squatting^38^.

In postindustrial environments, large-scale reductions in PA mirror increased LBP prevalence^2,39,40^. This association has led some to hypothesize that LBP is a consequence of mismatch conditions from the human body being poorly or inadequately adapted to novel environmental conditions, including ever reducing levels of PA^39,41–43^. Testing the mismatch hypothesis for LBP requires studying individuals in nonindustrial environments that engage more frequently in PAs like long-distance walking and carrying^44^. Because these populations live in remote, rural environments where OMCs are impractical for recording kinematics, IMUs are a promising alternative for measuring spine movement and tissue loading.

This study tests the accuracy of IMU-based estimates of lumbosacral (L5/S1) and thoracolumbar (T12/L1) joint 3D kinematics and kinetics during gait. For this, we compared IMU and OMC estimates of L5/S1 and T12/L1 3D joint angles, moments, and reaction forces using participant-specific Thoracolumbar Spine and Ribcage models during walking and running over three speeds each (six speeds total). We hypothesized all kinematic and kinetic estimates would not differ significantly between kinematic methods for any gait type at a given speed. We included both walking and running to test if the accuracy of IMUs holds regardless of activity intensity and type, as moments and reaction forces are expected to increase as speed increases, especially with the transition to running^7,45^. To test this assumption and further validate IMUs, we also report the effects of walking and running speed and method (IMU or OMC) on estimates of L5/S1 and T12/L1 3D joint moment and reaction force magnitudes.

## Methods

### Data collection

Five healthy adults (3 female, 2 male; age = 30 $\pm$ 5 yrs. (mean $\pm$ s.d.); body mass = 65.6 $\pm$ 9.3 kg; height = 1.73 $\pm$ 0.07 m) free of any MSK injuries or disorders participated in the experiment. Experimental procedures were conducted in the Skeletal Biology and Biomechanics Lab at Harvard University, Cambridge, MA. Participants provided their informed consent to a protocol approved by the Committee on the Use of Human Subjects at Harvard University. All methods were performed in accordance with the relevant guidelines and regulations.

### Motion measurement setup

**Figure 1:**
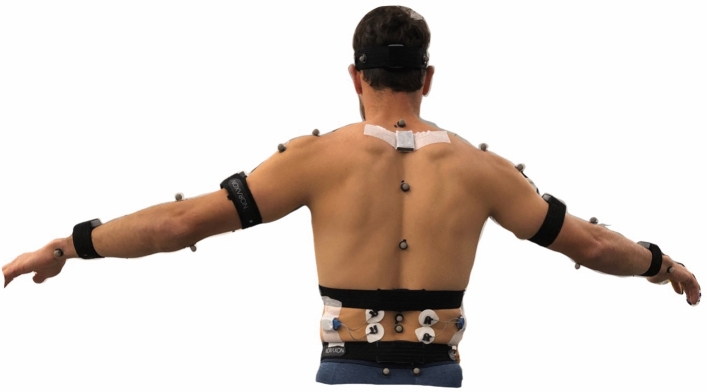
Photograph of participant wearing Qualisys reflective markers and Noraxon Ultium Motion inertial measurement units (IMU). Please see Methods for detailed description of marker and IMU placement. Data recorded from surface electromyography sensors on the lower back was not included in this study.

IMUs (Ultium Motion, Noraxon USA, Scottsdale, AZ, USA) were affixed to participants with either Velcro straps or double-sided adhesive tape over eight locations: the T2 spinous process, the L1 spinous process, pelvis (the body area of the sacrum), midway between both shoulder and elbow joints (on the lateral humeri), both posterior and distal forearms (where there is a low amount of muscle tissue), and the back of the head (Fig. 1). Prior to performing the data collection, the IMUs were calibrated to the signaling device with a correction for ferromagnetic disturbances due to electrical equipment in the laboratory.

In addition to IMUs, 27 infrared reflective markers (5 mm diameter) were affixed to participants’ skin and clothing with adhesive double-sided tape to the following bony landmarks: the spinous processes of C7, T4, T8, L2, and L3, sternoclavicular joint, xiphoid process, acromion processes, anterior and posterior superior iliac spines, medial and lateral humeral epicondyles, ulnar and radial styloid processes, bilateral frontal bone, bilateral occipital bone, and over the lateral deltoid muscles (Fig. 1).

### Experimental data collection

Following motion measurement setup, each participant completed seven unique static and dynamic activities over the course of one hour of testing. First, participants stood in a static pose for 10 s. Next, participants walked or ran at six different speeds for 60 s each. All conditions were performed on an instrumented double belt treadmill with force plates embedded (Bertec Corporation, Columbus, OH, USA) which measured GRFs. The GRFs were used to define bilateral heel strikes using a 20 N criteria and were synchronously recorded at 2,000 Hz within the OMC software. To ensure dynamic similarity of gait across different leg lengths, participant-specific gait speeds (m s^-1^) were calculated using the Froude (Fr) number:

1 $${\text{Fr}}=\frac{{speed}^{2}}{{\text{gl}}}$$

where *g* is gravitational acceleration (9.81 m s^−2^) and *l* is leg length, measured as the distance (m) from the greater trochanter of the right femur to the ground under the middle of the lateral foot during standing^46^. Fr numbers 0.1, 0.2, and 0.3 resulted in a walking gait. Fr numbers 0.6, 0.8, and 1.0 resulted in running gait. Participants first completed the walking conditions followed by running conditions, with the order of Fr number randomized within each gait. While participants performed each activity, IMU trajectories were sampled at 200 Hz (MR3.18, Noraxon USA, Scottsdale, AZ, USA), and OMC marker trajectories were sampled at 200 Hz using an eight-camera OMC system (Qualisys, Gothenburg, SWE). The IMU and OMC data were synchronously recorded via a hardware trigger.

### Musculoskeletal models

An OpenSim 4.4^17^ Thoracolumbar Spine and Ribcage model was used to estimate the kinematics and kinetics of the spine during walking and running. The full-body model includes 598 Hill-type muscle fascicles, 108 degrees of freedom, a fully articulated thoracolumbar spine (vertebrae T1-L5) with 3 rotational and 3 translational degrees of freedom at each joint, and ribcage (24 individual ribs plus a sternum)^22^. The model also includes a combined head and neck body and upper extremities. All major lumbar spine, thoracic spine, and abdominal muscle groups are incorporated. Trunk muscle cross-sectional areas and positions in the model were matched to measurements of 51 men (base male models) and 49 women (base female models) from the Framingham Heart Study^47^. The model has been previously validated for estimates of spine tissue loading and trunk muscle tension against indirect in vivo measurements of intradiscal pressure and vertebral compression force from telemeterized implants^12^.

Participant-specific models were created for both IMU and OMC using the OpenSim Scaling Tool, which adjusted the default height and body mass of gender-matched base models to match each participant’s measured height and body mass. For OMC-created models only, the OpenSim Scaling Tool additionally adjusted model marker positions based on marker positions in the standing static trial.

### Spine kinematic analysis

For the IMU data, 3D spine segment (lumbar, thoracic, and cervical) and upper extremity joint angle outputs from the recorded motions in Noraxon MR3.18 software were converted into an OpenSim-compatible inverse kinematics motion file using custom MATLAB (The MathWorks, Inc., Natick, MA, USA) scripts. Because the pelvis was the base (most inferior) segment modeled in Noraxon MR3.18 software, in the absence of foot IMUs, pelvis positions were fixed in space. However, pelvis displacement during walking and running affects spine kinetics. Therefore, we estimated pelvis position for IMU models in the OpenSim world frame by double integrating the pelvis IMU accelerations. Prior to integration, we applied a low-pass, zero-phase, second-order 30 Hz Butterworth filter to the pelvis IMU acceleration signal. Following the integration, we applied a high-pass, zero-phase 1 Hz Butterworth filter to the integrated signal to remove linear drift.

For OMC data, the OpenSim Inverse Kinematics Tool calculated the coordinates of each body segment during the recorded motions using a least-squares method to minimize the difference between the recorded marker positions and their location on the model while accounting for the kinematic constraints of the model^48^. Marker data were not filtered, however resulting inverse kinematics coordinates were filtered with a low-pass, zero-phase, fourth-order 4 Hz Butterworth filter prior to performing kinetic analyses, which was determined via both residual analysis and Fourier analysis of both IMU and OMC kinematic time series data^49^.

### Spine kinetic analysis

The OpenSim models used a top-down modeling approach to evaluate the spine, with segment analysis commencing at the distal segments (e.g. hands) and working down through the kinetic chain to the lumbosacral joint. The OpenSim Inverse Dynamics Tool calculated 3D joint moments in flexion–extension (FE), lateral bending (LB), and axial rotation (AR) at both the L5/S1 and T12/L1 joints from the IMU and OMC inverse kinematics coordinates. The OpenSim Static Optimization Tool estimated the model muscle forces that can produce the calculated joint moments by minimizing the total cubed activations of all musculotendon actuators. Finally, the OpenSim Joint Reaction Analysis Tool was used to calculate the combined effect of reactionary and muscle forces on the L5/S1 and T12/L1 compression, anteroposterior (AP) shear, and mediolateral (ML) shear forces. For OMC analyses, we applied the measured GRFs to the pelvis, the base segment of the OpenSim models, as part of the “gold standard” modeling approach. Applying GRFs to the pelvis reduced residual actuator forces but was not necessary for estimating kinetics with this top-down model. We did not apply the measured GRFs to the pelvis for IMU analyses because we were interested in validating IMUs for non-lab, real-world environments where GRFs are often unavailable.

### Statistical analysis

The dependent variables for IMU and OMC kinematics and kinetics were L5/S1 and T12/L1 3D joint angles, moments, and reaction forces. Joint moments and reaction forces were made non-dimensional by dividing by the product of body weight and height and by body weight, respectively. To compare IMU and OMC dependent variables, time series were ensemble averaged over full strides (heel strike to ipsilateral heel strike). Joint angles were referenced relative to the first initial heel contact value of the ensemble average from all subsequent values, thus setting the first value to zero degrees. Per gait condition, an average of 79 strides were used for analysis across the five participants.

Root mean squared errors (*RMSE*) between the mean value of the IMU and OMC ensemble averages for each variable ($\overline{{\text{imu}} }$ and $\overline{{\text{omc}} }$) quantified the difference between the signals. Normalized *RMSE*s (*NRMSE*) further quantified differences relative to the range (maximum minus minimum) of the OMC signal:

2 $$\mathrm{RMSE }= \sqrt{\sum {(\overline{{\text{imu}} } - \overline{{\text{omc}} })}^{2}}$$

3 $$\mathrm{NRMSE }= \frac{\sqrt{\sum {(\overline{{\text{imu}} } - \overline{{\text{omc}} })}^{2}}}{{\overline{{\text{omc}}} }_{{\text{max}}} - {\overline{{\text{omc}}} }_{{\text{min}}}}$$

Temporal association was computed as the cross-correlation (*R*) between IMU and OMC ensemble-averaged trajectories. Cross-correlations measure the similarity between trajectories as a function of lag, defined here as percent (%) of the stride cycle. Interpretation of *R* effect size was based on Cohen’s criteria (0.10 = small effect; 0.30 = medium; 0.50 = large)^50^.

To test the extent to which running involves higher magnitude spine joint moments and reaction forces than walking and whether IMU estimates of spine joint moment and reaction force magnitudes differed from OMC estimates, nonparametric Friedman’s tests were performed on the root mean squares (*RMS*) of IMU and OMC ensemble averaged L5/S1 and T12/L1 kinetic variables for each condition and method. *RMS* values were used to correct for the magnitudes switching signs halfway through a stride. Nonparametric tests were used because q-q plots showed residuals of the *RMS* values were not normally distributed.

All significant ($\alpha$ = 0.05) Friedman’s test effects were evaluated post hoc with Wilcoxon signed-rank tests with Bonferroni corrections to account for multiple pairwise comparisons ($\alpha$ = 0.00 $\overline{3 }$; six conditions with 15 comparisons)^51^. Statistical analyses were performed in custom MATLAB scripts using The Statistics and Machine Learning Toolbox.

## Results

The average leg length of participants was 0.93 $\pm$ 0.04 m (mean $\pm$ s.d.). Walking at Fr numbers 0.1, 0.2, and 0.3 corresponded to speeds of 0.95 $\pm$ 0.02, 1.35 $\pm$ 0.03, and 1.65 $\pm$ 0.04 m s^-1^, respectively. Running at Fr numbers 0.6, 0.8, and 1.0 corresponded to speeds of 2.34 $\pm$ 0.06, 2.70 $\pm$ 0.06, and 3.02 $\pm$ 0.07 m s^−1^, respectively.

### Joint angle, moment, and reaction force comparisons between IMU and OMC

**Figure 2:**
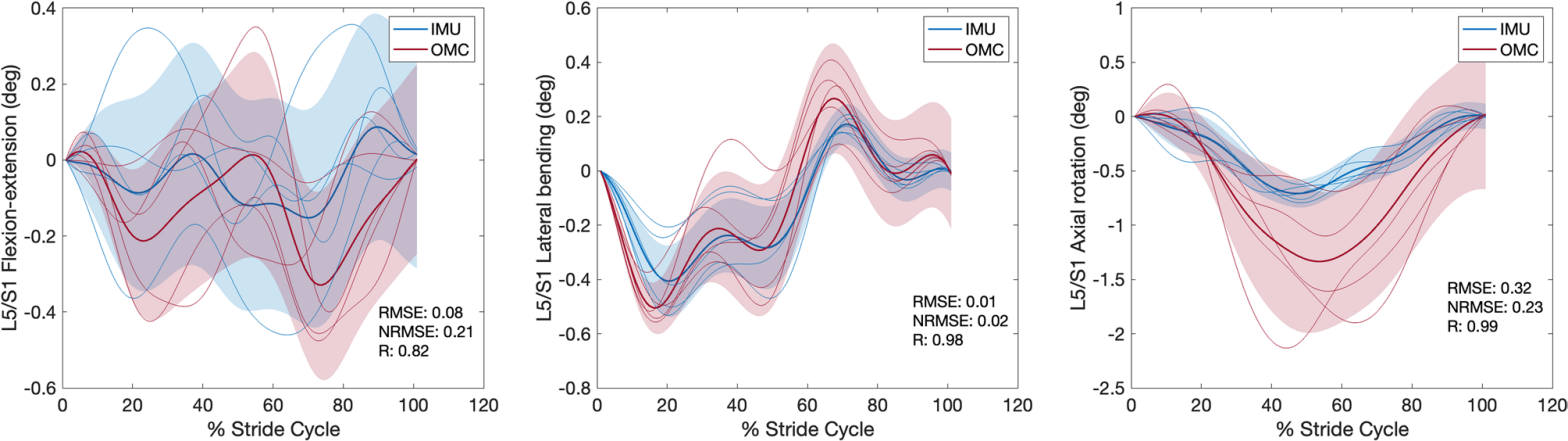
Lumbosacral (L5/S1) joint angles (referenced to initial heel strike value) during walking at Froude number = 0.2. Thick lines are means of ensemble averages, with shaded region as the standard deviation. Thin lines are means of participant ensemble averages. IMU = inertial measurement units; OMC = optical motion capture; *RMSE* = root mean squared error; *NRMSE* = normalized root mean squared error; *R* = cross-correlation.

**Figure 3:**
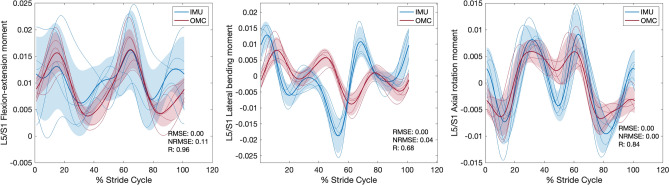
Lumbosacral (L5/S1) joint moments (normalized to body weight times height) during walking at Froude number = 0.2. Thick lines are means of ensemble averages, with shaded region as the standard deviation. Thin lines are means of participant ensemble averages. IMU = inertial measurement units; OMC = optical motion capture; *RMSE* = root mean squared error; *NRMSE* = normalized root mean squared error; *R* = cross-correlation.

**Figure 4:**
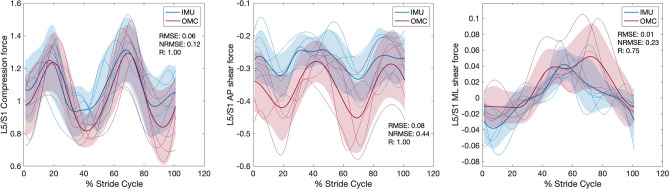
Lumbosacral (L5/S1) joint reaction forces (normalized to body weight) during walking at Froude number = 0.2. Thick lines are means of ensemble averages, with shaded region as the standard deviation. Thin lines are means of participant ensemble averages. IMU = inertial measurement units; OMC = optical motion capture; AP = anteroposterior; ML = mediolateral; *RMSE* = root mean squared error; *NRMSE* = normalized root mean squared error; *R* = cross-correlation.

**Figure 5:**
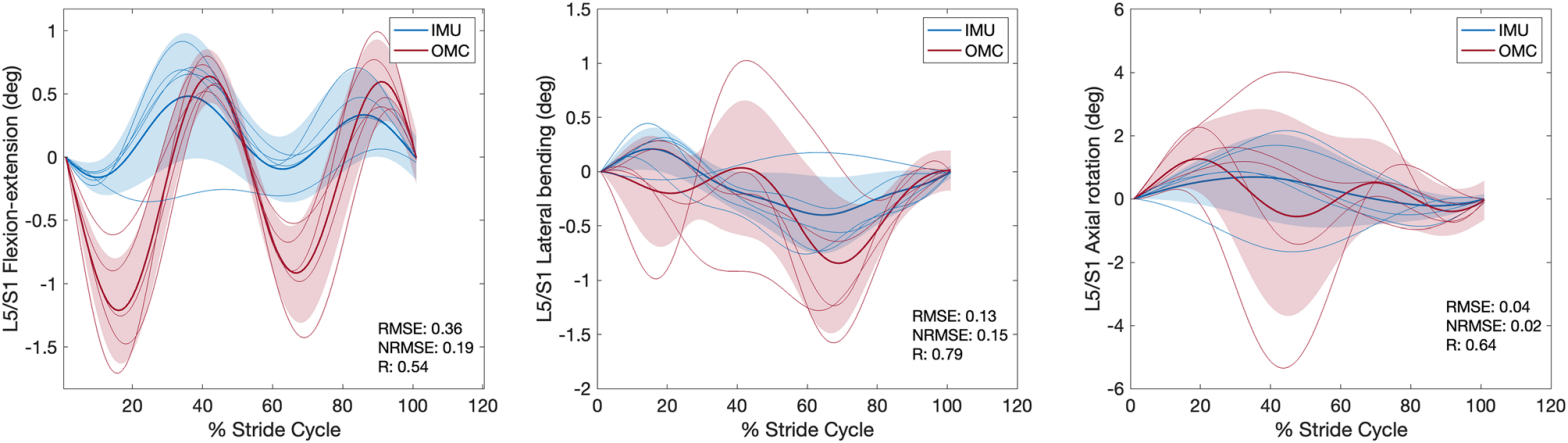
Lumbosacral (L5/S1) joint angles (referenced to initial heel strike value) during running at Froude number = 0.8. Thick lines are means of ensemble averages, with shaded region as the standard deviation. Thin lines are means of participant ensemble averages. IMU = inertial measurement units; OMC = optical motion capture; *RMSE* = root mean squared error; *NRMSE* = normalized root mean squared error; *R* = cross-correlation.

**Figure 6:**
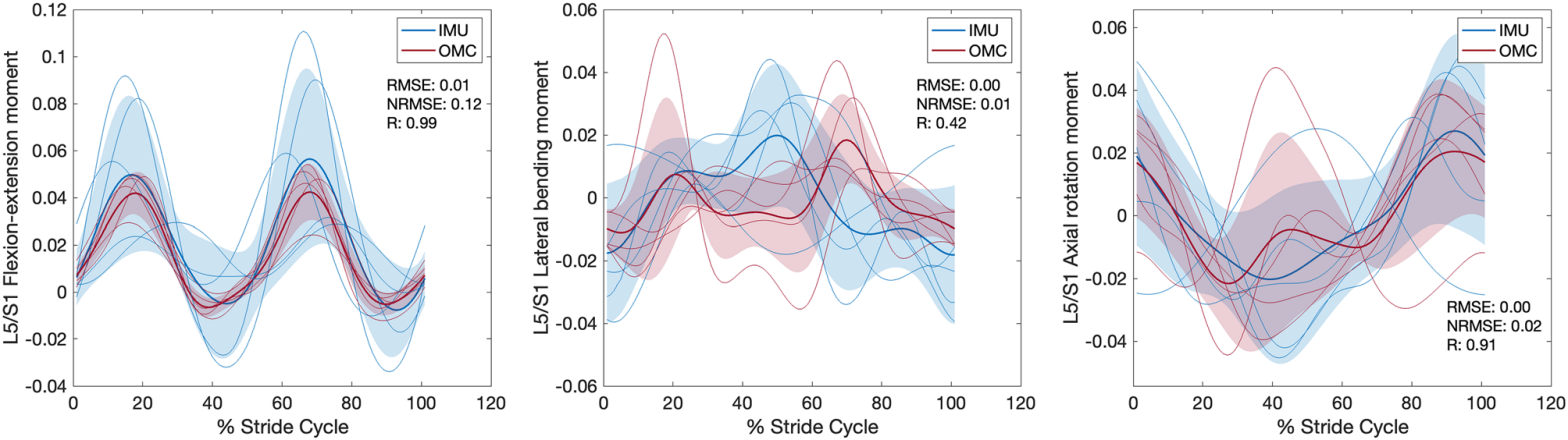
Lumbosacral (L5/S1) joint moments (normalized to body weight times height) during running at Froude number = 0.8. Thick lines are means of ensemble averages, with shaded region as the standard deviation. Thin lines are means of participant ensemble averages. IMU = inertial measurement units; OMC = optical motion capture; *RMSE* = root mean squared error; *NRMSE* = normalized root mean squared error; *R* = cross-correlation.

**Figure 7:**
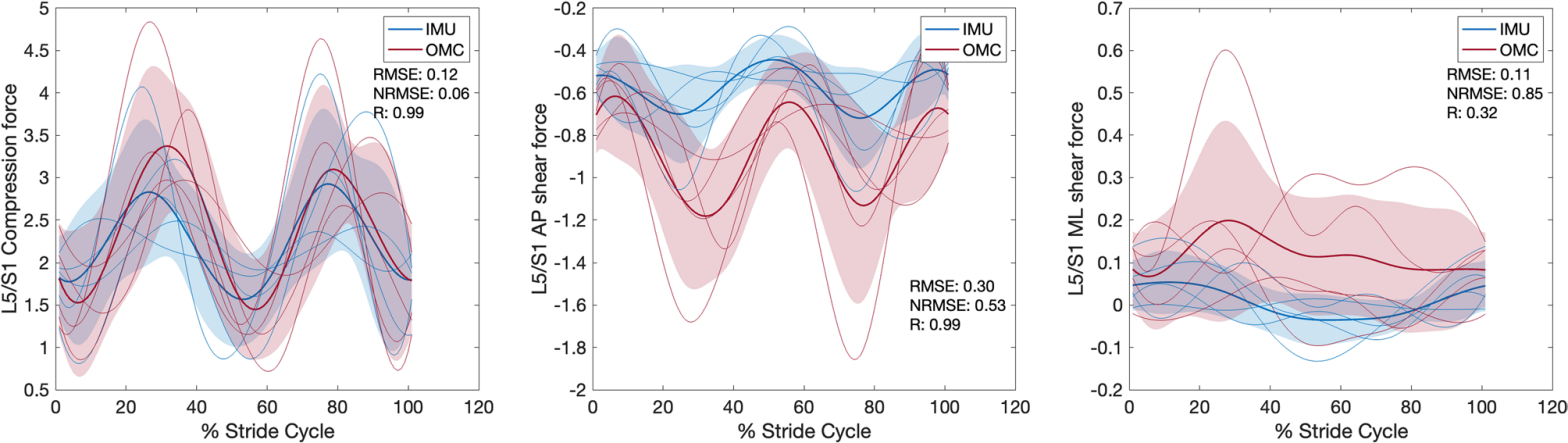
Lumbosacral (L5/S1) joint reaction forces (normalized to body weight) during running at Froude number = 0.8. Thick lines are means of ensemble averages, with shaded region as the standard deviation. Thin lines are means of participant ensemble averages. IMU = inertial measurement units; OMC = optical motion capture; AP = anteroposterior; ML = mediolateral; *RMSE* = root mean squared error; *NRMSE* = normalized root mean squared error; *R* = cross-correlation.

For each condition, *RMSE*, *NRMSE*, *R* values, and % stride cycle lags for IMU and OMC estimated 3D joint angles, moments, and reaction forces are presented in Tables 1, 2, and 3, respectively. Line plots for one representative walking condition (Fr 0.2) comparing L5/S1 IMU and OMC estimated 3D joint angles, moments, and reaction forces are presented in Figs. 2, 3, and 4. Line plots for one representative running condition (Fr 0.8) comparing L5/S1 IMU and OMC estimated 3D joint angles, moments, and reaction forces are presented in Figs. 5, 6, and 7. Across all conditions and both joints, *NRMSE* values for IMU and OMC estimated 3D joint angle, moment, and reaction force comparisons were generally low (< 0.3 for 81% of comparisons). *NRMSE* values for comparisons involving AP and ML shear forces were generally larger than other dependent kinetic variables (> 0.3 for 50% of comparisons). Cross-correlations between IMU and OMC estimated 3D joint angles, moments, and reaction forces were generally positive and high (> 0.5 for 91% of comparisons), with few exceptions.

**Table 1:** Joint angle (referenced to initial heel strike value) comparisons between inertial measurement units and optical motion capture.

Froude	Axis	L5/S1				T12/L1			
		*RMSE*	*NRMSE*	*R*	Lag	*RMSE*	*NRMSE*	*R*	Lag
0.1 (Walk)	FE	0.08	0.21	0.46	25	0.02	0.21	0.46	25
	LB	0.03	0.06	0.91	3	0.05	0.24	0.86	0
	AR	0.30	0.29	0.99	− 6	0.02	0.12	0.99	3
0.2 (Walk)	FE	0.08	0.21	0.82	− 12	0.02	0.21	0.82	− 12
	LB	0.01	0.02	0.98	4	0.05	0.14	0.93	7
	AR	0.32	0.23	0.99	− 3	0.01	0.03	0.99	2
0.3 (Walk)	FE	0.08	0.26	0.77	− 15	0.02	0.26	0.77	− 15
	LB	0.02	0.02	0.98	5	0.10	0.23	0.94	12
	AR	0.43	0.25	1.00	− 3	0.02	0.09	1.00	5
0.6 (Run)	FE	0.25	0.18	0.69	19	0.07	0.18	0.69	19
	LB	0.19	0.35	0.15	− 32	0.47	0.37	0.83	1
	AR	0.00	0.00	0.86	0	0.05	0.26	0.92	− 16
0.8 (Run)	FE	0.36	0.19	0.54	20	0.10	0.19	0.54	20
	LB	0.13	0.15	0.79	− 6	0.37	0.24	0.02	28
	AR	0.04	0.02	0.64	21	0.12	0.23	0.94	− 14
1.0 (Run)	FE	0.25	0.17	0.75	24	0.07	0.17	0.75	24
	LB	0.32	0.32	0.25	11	0.58	0.34	0.06	− 28
	AR	1.32	0.37	0.99	− 1	0.22	0.65	0.02	− 10

L5/S1, lumbosacral joint; T12/L1,  thoracolumbar joint; FE, flexion–extension; LB, lateral bending; AR, axial rotation; *RMSE*, root mean squared error; *NRMSE*, normalized root mean squared error; *R* , cross-correlation. Lag is % of stride cycle (positive value indicates OMC signal occurs before IMU signal).

**Table 2:** Joint moment (normalized to body weight times height) comparisons between inertial measurement units and optical motion capture.

Froude	Axis	L5/S1				T12/L1			
		*RMSE*	*NRMSE*	*R*	Lag	*RMSE*	*NRMSE*	*R*	Lag
0.1 (Walk)	FE	< 0.01	0.20	0.96	0	< 0.01	0.18	0.99	0
	LB	< 0.01	0.00	0.61	6	< 0.01	0.02	0.57	− 19
	AR	< 0.01	0.01	0.95	0	< 0.01	0.02	0.87	− 5
0.2 (Walk)	FE	< 0.01	0.11	0.96	0	< 0.01	0.11	0.99	0
	LB	< 0.01	0.04	0.68	8	< 0.01	0.05	0.72	− 16
	AR	< 0.01	< 0.01	0.84	2	< 0.01	0.00	0.94	− 4
0.3 (Walk)	FE	< 0.01	0.07	0.91	0	< 0.01	0.08	0.98	0
	LB	< 0.01	0.01	0.76	-9	< 0.01	0.01	0.80	− 11
	AR	< 0.01	0.01	0.86	3	< 0.01	0.01	0.90	− 5
0.6 (Run)	FE	0.01	0.16	0.99	2	< 0.01	0.14	0.99	1
	LB	< 0.01	< 0.01	0.62	− 2	< 0.01	0.13	0.69	− 6
	AR	< 0.01	0.01	0.91	2	< 0.01	0.01	0.94	0
0.8 (Run)	FE	0.01	0.12	0.99	1	< 0.01	0.10	0.97	0
	LB	< 0.01	0.01	0.42	− 23	< 0.01	0.06	0.66	− 7
	AR	< 0.01	0.02	0.91	0	< 0.01	0.01	0.91	0
1.0 (Run)	FE	0.01	0.11	0.99	3	< 0.01	0.11	0.99	2
	LB	< 0.01	0.02	0.67	− 2	< 0.01	0.02	0.77	− 8
	AR	< 0.01	0.03	0.91	0	< 0.01	0.00	0.94	0

L5/S1, lumbosacral joint; T12/L1, thoracolumbar joint; FE, flexion–extension; LB, lateral bending; AR, axial rotation; *RMSE*, root mean squared error; *NRMSE*, normalized root mean squared error; *R*, cross-correlation. Lag is % of stride cycle (positive value indicates OMC signal occurs before IMU signal).

**Table 3:** Joint reaction force (normalized to body weight) comparisons between inertial measurement units and optical motion capture.

Froude	Axis	L5/S1				T12/L1			
		*RMSE*	*NRMSE*	*R*	Lag	*RMSE*	*NRMSE*	*R*	Lag
0.1 (Walk)	Comp	0.01	0.05	1.00	0	0.06	0.32	1.00	0
	AP shear	0.10	1.15	1.00	0	< 0.01	0.01	1.00	0
	ML shear	0.03	0.51	0.59	− 10	< 0.01	0.02	0.81	0
0.2 (Walk)	Comp	0.06	0.12	1.00	0	0.08	0.22	0.99	0
	AP shear	0.08	0.44	1.00	0	< 0.01	0.01	1.00	0
	ML shear	0.01	0.23	0.75	− 3	< 0.01	0.02	0.63	1
0.3 (Walk)	Comp	0.05	0.08	0.99	0	0.07	0.14	0.98	0
	AP shear	0.09	0.47	1.00	0	< 0.01	0.02	1.00	0
	ML shear	0.02	0.19	0.75	− 12	< 0.01	0.00	0.58	− 4
0.6 (Run)	Comp	0.06	0.03	1.00	0	0.14	0.11	1.00	0
	AP shear	0.27	0.46	0.99	0	0.01	0.26	0.85	0
	ML shear	0.08	0.74	0.61	− 32	0.01	0.10	0.82	− 1
0.8 (Run)	Comp	0.12	0.06	0.99	0	0.08	0.07	0.99	0
	AP shear	0.30	0.53	0.99	0	0.02	0.36	0.95	0
	ML shear	0.11	0.85	0.32	30	0.02	0.10	0.81	0
1.0 (Run)	Comp	0.15	0.06	0.98	0	0.25	0.15	0.98	− 2
	AP shear	0.25	0.30	0.99	0	0.04	0.34	0.87	0
	ML shear	0.12	1.50	0.54	31	0.04	0.32	0.37	19

L5/S1, lumbosacral joint; T12/L1, thoracolumbar joint; Comp, compression; AP, anteroposterior; ML, mediolateral; *RMSE*, root mean squared error; *NRMSE*, normalized root mean squared error; *R*, cross-correlation. Lag is % of stride cycle (positive value indicates OMC signal occurs before IMU signal).

### Effect of walking and running speed and method on joint moments and reaction forces

**Figure 8:**
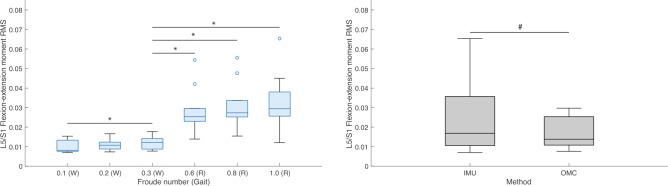
Boxplots of lumbosacral (L5/S1) joint root mean squares (*RMS*) of flexion–extension moments (normalized to the product of body weight and height) by condition (left) and method (right). W = Walk; R = Run. * indicates *P* value < 0.00 $\overline{3 }$. ^#^ indicates *P* value < 0.05. All R conditions significantly different from 0.3 (W) are also different from 0.1 (W) and 0.2 (W). IMU = inertial measurement units; OMC = optical motion capture.

**Figure 9:**
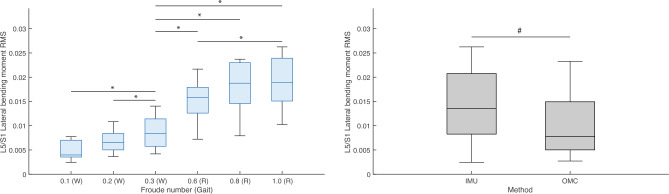
Boxplots of lumbosacral (L5/S1) joint root mean squares (*RMS*) of lateral bending moments (normalized to the product of body weight and height) by condition (left) and method (right). W = Walk; R = Run. * indicates *P* value < 0.00 $\overline{3 }$. ^#^ indicates *P* value < 0.05. All R conditions significantly different from 0.3 (W) are also different from 0.1 (W) and 0.2 (W). IMU = inertial measurement units; OMC = optical motion capture.

**Figure 10:**
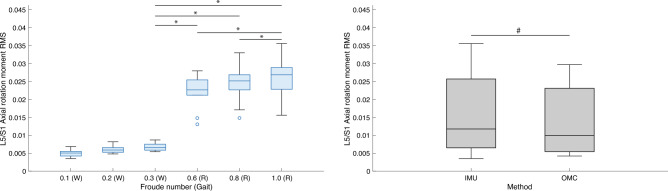
Boxplots of lumbosacral (L5/S1) joint root mean squares (*RMS*) of axial rotation moments (normalized to the product of body weight and height) by condition (left) and method (right). W = Walk; R = Run. * indicates *P* value < 0.00 $\overline{3 }$. ^#^ indicates *P* value < 0.05. All R conditions significantly different from 0.3 (W) are also different from 0.1 (W) and 0.2 (W). IMU = inertial measurement units; OMC = optical motion capture.

**Figure 11:**
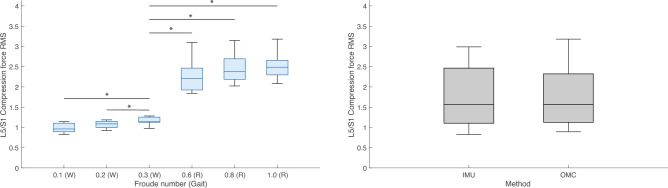
Boxplots of lumbosacral (L5/S1) joint root mean squares (*RMS*) of compression forces (normalized to body weight) by condition (left) and method (right). W = Walk; R = Run. * indicates *P* value < 0.00 $\overline{3 }$. All R conditions significantly different from 0.3 (W) are also different from 0.1 (W) and 0.2 (W). IMU = inertial measurement units; OMC = optical motion capture.

**Figure 12:**
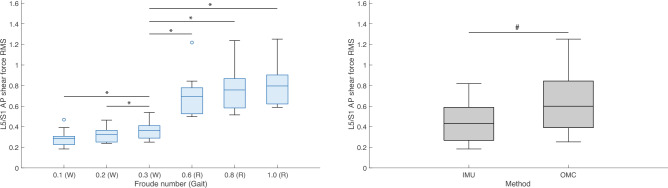
Boxplots of lumbosacral (L5/S1) joint root mean squares (*RMS*) of anteroposterior (AP) shear forces (normalized to body weight) by condition (left) and method (right). W = Walk; R = Run. * indicates *P* value < 0.00 $\overline{3 }$. ^#^ indicates *P* value < 0.05. All R conditions significantly different from 0.3 (W) are also different from 0.1 (W) and 0.2 (W). IMU = inertial measurement units; OMC = optical motion capture.

**Figure 13:**
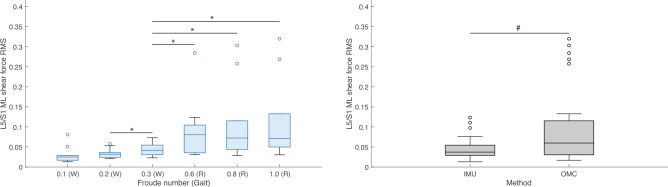
Boxplots of lumbosacral (L5/S1) joint root mean squares (*RMS*) of mediolateral (ML) shear forces (normalized to body weight) by condition (left) and method (right). W = Walk; R = Run. * indicates *P* value < 0.00 $\overline{3 }$. ^#^ indicates *P* value < 0.05. All R conditions significantly different from 0.3 (W) are also different from 0.1 (W) and 0.2 (W). IMU = inertial measurement units; OMC = optical motion capture.

Friedman’s tests were performed to test the effect of condition (Fr number) and method (IMU or OMC) on the *RMS* values of ensemble averaged L5/S1 and T12/L1 3D joint moments and reaction forces (hereafter, “*RMS*”). Means and standard deviations of *RMS* joint moments by condition are presented in Table 4. Percentage differences and results of post hoc tests for the effect of condition on *RMS* joint moments are presented in Table 5. Means and standard deviations of *RMS* joint moments by method are presented in Table 6. Percentage differences and results of post hoc tests for the effect of method on *RMS* joint moments are presented in Table 7. Means and standard deviations of *RMS* joint reaction forces by condition are presented in Table 8. Percentage differences and results of post hoc tests for the effect of condition on *RMS* joint reaction forces are presented in Table 9. Means and standard deviations of *RMS* joint reaction forces by method are presented in Table 10. Percentage differences and results of post hoc tests for the effect of method on *RMS* joint reaction forces are presented in Table 11. Boxplots of L5/S1 *RMS* joint moments in FE, LB, and AR by condition and method are presented in Figs. 8, 9, and 10, respectively. Boxplots of L5/S1 *RMS* joint reaction forces in compression, AP shear, and ML shear by condition and method are presented in Figs. 11, 12, and 13. Friedman’s test summary statistics are presented in SI Tables 1, 2, 3 and 4.

**Table 4:** Means and standard deviations (S.D.) of root mean squares of ensemble averaged lumbosacral (L5/S1) and thoracolumbar (T12/L1) 3D joint moments (normalized to body weight times height) by Froude number.

Froude	Axis	L5/S1	T12/L1
		Mean (S.D.)	Mean (S.D.)
0.1 (Walk)	FE	0.010 (0.003)	0.009 (0.002)
	LB	0.005 (0.002)	0.003 (0.001)
	AR	0.005 (0.001)	0.004 (0.001)
0.2 (Walk)	FE	0.011 (0.003)	0.009 (0.002)
	LB	0.007 (0.002)	0.003 (0.001)
	AR	0.006 (0.001)	0.005 (0.001)
0.3 (Walk)	FE	0.012 (0.003)	0.009 (0.002)
	LB	0.009 (0.003)	0.004 (0.001)
	AR	0.007 (0.001)	0.006 (0.001)
0.6 (Run)	FE	0.028 (0.012)	0.020 (0.007)
	LB	0.016 (0.005)	0.009 (0.003)
	AR	0.022 (0.005)	0.018 (0.005)
0.8 (Run)	FE	0.030 (0.013)	0.021 (0.007)
	LB	0.018 (0.005)	0.009 (0.002)
	AR	0.024 (0.005)	0.020 (0.005)
1.0 (Run)	FE	0.033 (0.014)	0.022 (0.008)
	LB	0.019 (0.006)	0.010 (0.003)
	AR	0.026 (0.006)	0.022 (0.006)

FE = flexion–extension; LB = lateral bending; AR = axial rotation.

**Table 5:** Post hoc tests and percent differences regarding the effect of Froude number on root mean squares of lumbosacral (top section) and thoracolumbar (bottom section) joint moments. Post hoc tests were nonparametric Wilcoxon signed-rank tests. % diff calculated as ((Froude A – Froude B)/Froude B)*100.

Froude A	Froude B	FE		LB		AR	
		% diff	*P*	% diff	*P*	% diff	*P*
0.1 (Walk)	0.2 (Walk)	− 11.8	0.020	− 31.5	0.010	− 17.6	0.020
0.1 (Walk)	0.3 (Walk)	− 18.1	0.002	− 46.5	0.002	− 25.2	0.014
0.1 (Walk)	0.6 (Run)	− 64.9	0.002	− 69.4	0.002	− 76.7	0.002
0.1 (Walk)	0.8 (Run)	− 67.4	0.002	− 73.2	0.002	− 78.9	0.002
0.1 (Walk)	1.0 (Run)	− 69.8	0.002	− 75.0	0.002	− 80.2	0.002
0.2 (Walk)	0.3 (Walk)	− 7.2	0.027	− 21.8	0.002	− 9.2	0.037
0.2 (Walk)	0.6 (Run)	− 60.2	0.002	− 55.3	0.002	− 71.8	0.002
0.2 (Walk)	0.8 (Run)	− 63.1	0.002	− 60.8	0.002	− 74.4	0.002
0.2 (Walk)	1.0 (Run)	− 65.8	0.002	− 63.5	0.002	− 75.9	0.002
0.3 (Walk)	0.6 (Run)	− 57.1	0.002	− 42.8	0.002	− 68.9	0.002
0.3 (Walk)	0.8 (Run)	− 60.2	0.002	− 49.9	0.002	− 71.8	0.002
0.3 (Walk)	1.0 (Run)	− 63.1	0.002	− 53.3	0.002	− 73.5	0.002
0.6 (Run)	0.8 (Run)	− 7.3	0.010	− 12.4	0.010	− 9.4	0.004
0.6 (Run)	1.0 (Run)	− 14.0	0.004	− 18.3	0.002	− 14.7	0.002
0.8 (Run)	1.0 (Run)	− 7.2	0.160	− 6.8	0.020	− 5.8	0.002
0.1 (Walk)	0.2 (Walk)	− 5.6	0.160	− 10.4	0.160	− 27.6	0.010
0.1 (Walk)	0.3 (Walk)	− 8.1	0.027	− 33.1	0.004	− 38.3	0.004
0.1 (Walk)	0.6 (Run)	− 56.7	0.002	− 68.6	0.002	− 79.8	0.002
0.1 (Walk)	0.8 (Run)	− 59.3	0.002	− 69.9	0.002	− 81.9	0.002
0.1 (Walk)	1.0 (Run)	− 61.2	0.002	− 73.3	0.002	− 83.0	0.002
0.2 (Walk)	0.3 (Walk)	− 2.7	0.322	− 25.3	0.004	− 14.8	0.002
0.2 (Walk)	0.6 (Run)	− 54.1	0.002	− 64.9	0.002	− 72.0	0.002
0.2 (Walk)	0.8 (Run)	− 56.8	0.002	− 66.4	0.002	− 75.0	0.002
0.2 (Walk)	1.0 (Run)	− 58.9	0.002	− 70.2	0.002	− 76.6	0.002
0.3 (Walk)	0.6 (Run)	− 52.9	0.002	− 53.0	0.002	− 67.2	0.002
0.3 (Walk)	0.8 (Run)	− 55.7	0.002	− 55.0	0.002	− 70.7	0.002
0.3 (Walk)	1.0 (Run)	− 57.8	0.002	− 60.1	0.002	− 72.5	0.002
0.6 (Run)	0.8 (Run)	− 5.9	0.014	− 4.2	0.105	− 10.7	0.002
0.6 (Run)	1.0 (Run)	− 10.4	0.004	− 15.0	0.064	− 16.2	0.002
0.8 (Run)	1.0 (Run)	− 4.8	0.232	− 11.3	0.004	− 6.1	0.002

$\alpha = 0.00\overline{3 }$ for 15 comparisons across methods. FE, flexion–extension; LB, lateral bending; AR, axial rotation.

**Table 6:** Means and standard deviations (S.D.) of root mean squares of ensemble averaged lumbosacral (L5/S1) and thoracolumbar (T12/L1) 3D joint moments (normalized to body weight times height) by method (inertial measurement unit [IMU] or optical motion capture [OMC]) across conditions.

Method	L5/S1			T12/L1		
	FE	LB	AR	FE	LB	AR
	Mean (S.D.)	Mean (S.D.)	Mean (S.D.)	Mean (S.D.)	Mean (S.D.)	Mean (S.D.)
IMU	0.024 (0.017)	0.014 (0.007)	0.016 (0.010)	0.017 (0.009)	0.005 (0.003)	0.014 (0.010)
OMC	0.017 (0.001)	0.010 (0.006)	0.014 (0.009)	0.013 (0.005)	0.007 (0.004)	0.011 (0.007)

FE, flexion–extension; LB, lateral bending; AR, axial rotation..

**Table 7:** Nonparametric Friedman’s tests and percent differences regarding the effect of method (inertial measurement unit [IMU] or optical motion capture [OMC]) on root mean squares of lumbosacral (L5/S1) and thoracolumbar (T12/L1) joint moments across conditions.

Joint	FE		LB		AR	
	% diff	*P*	% diff	*P*	% diff	*P*
L5/S1	42.2	0.009	42.7	< 0.001	13.2	0.001
T12/L1	29.9	0.046	− 25.6	0.001	23.1	0.162

% diff calculated as ((IMU–OMC)/OMC)*100. $\alpha = 0.05$. FE, flexion–extension; LB, lateral bending; AR,  axial rotation.

**Table 8:** Means and standard deviations (S.D.) of root mean squares of ensemble averaged lumbosacral (L5/S1) and thoracolumbar (T12/L1) 3D joint reaction forces (normalized to body weight) by Froude number.

Froude	Axis	L5/S1	T12/L1
		Mean (S.D.)	Mean (S.D.)
0.1 (Walk)	Comp	0.992 (0.121)	0.665 (0.078)
	AP shear	0.291 (0.086)	0.111 (0.001)
	ML shear	0.030 (0.021)	0.024 (0.007)
0.2 (Walk)	Comp	1.076 (0.088)	0.711 (0.064)
	AP shear	0.322 (0.075)	0.109 (0.012)
	ML shear	0.034 (0.013)	0.031 (0.009)
0.3 (Walk)	Comp	1.165 (0.095)	0.762 (0.064)
	AP shear	0.361 (0.088)	0.112 (0.018)
	ML shear	0.045 (0.016)	0.036 (0.009)
0.6 (Run)	Comp	2.274 (0.397)	1.398 (0.260)
	AP shear	0.710 (0.219)	0.103 (0.061)
	ML shear	0.092 (0.076)	0.086 (0.015)
0.8 (Run)	Comp	2.436 (0.351)	1.474 (0.235)
	AP shear	0.770 (0.216)	0.099 (0.049)
	ML shear	0.110 (0.095)	0.093 (0.017)
1.0 (Run)	Comp	2.520 (0.353)	1.523 (0.241)
	AP shear	0.799 (0.204)	0.105 (0.045)
	ML shear	0.116 (0.100)	0.102 (0.020)

Comp, compression; AP, anteroposterior; ML,  mediolateral.

**Table 9:** Post hoc tests and percent differences regarding the effect of Froude number on root mean squares of lumbosacral (top section) and thoracolumbar (bottom section) joint reaction forces. Post hoc tests were nonparametric Wilcoxon signed-rank tests.

Froude A	Froude B	Comp		AP shear		ML shear	
		% diff	*P*	% diff	*P*	% diff	*P*
0.1 (Walk)	0.2 (Walk)	− 7.9	0.006	− 9.8	0.004	− 12.3	0.084
0.1 (Walk)	0.3 (Walk)	− 14.9	0.002	− 19.4	0.002	− 33.2	0.037
0.1 (Walk)	0.6 (Run)	− 56.4	0.002	− 59.0	0.002	− 67.4	0.002
0.1 (Walk)	0.8 (Run)	− 59.3	0.002	− 62.2	0.002	− 72.8	0.002
0.1 (Walk)	1.0 (Run)	− 60.6	0.002	− 63.6	0.002	− 74.3	0.002
0.2 (Walk)	0.3 (Walk)	− 7.5	0.002	− 10.6	0.002	− 23.8	0.002
0.2 (Walk)	0.6 (Run)	− 52.6	0.002	− 54.6	0.002	− 62.9	0.010
0.2 (Walk)	0.8 (Run)	− 55.8	0.002	− 58.1	0.002	− 68.9	0.006
0.2 (Walk)	1.0 (Run)	− 57.3	0.002	− 59.6	0.002	− 70.7	0.004
0.3 (Walk)	0.6 (Run)	− 48.8	0.002	− 49.2	0.002	− 51.3	0.037
0.3 (Walk)	0.8 (Run)	− 52.2	0.002	− 53.1	0.002	− 59.3	0.027
0.3 (Walk)	1.0 (Run)	− 53.8	0.002	− 54.9	0.002	− 61.5	0.014
0.6 (Run)	0.8 (Run)	− 6.7	0.006	− 7.8	0.004	− 16.4	0.084
0.6 (Run)	1.0 (Run)	− 9.8	0.004	− 11.1	0.004	− 21.1	0.064
0.8 (Run)	1.0 (Run)	− 3.3	0.193	− 3.6	0.064	− 5.6	0.064
0.1 (Walk)	0.2 (Walk)	− 6.4	0.010	2.4	0.275	− 20.9	0.064
0.1 (Walk)	0.3 (Walk)	− 12.7	0.002	− 0.9	0.846	− 32.6	0.002
0.1 (Walk)	0.6 (Run)	− 52.5	0.002	8.2	0.232	− 71.5	0.002
0.1 (Walk)	0.8 (Run)	− 54.9	0.002	12.1	0.322	− 73.8	0.002
0.1 (Walk)	1.0 (Run)	− 56.4	0.002	6.5	0.275	− 76.0	0.002
0.2 (Walk)	0.3 (Walk)	− 6.7	0.002	− 3.2	0.232	− 14.8	0.010
0.2 (Walk)	0.6 (Run)	− 49.2	0.002	5.6	0.322	− 64.0	0.002
0.2 (Walk)	0.8 (Run)	− 51.8	0.002	9.4	0.322	− 66.9	0.002
0.2 (Walk)	1.0 (Run)	− 53.3	0.002	4.0	0.375	− 69.7	0.002
0.3 (Walk)	0.6 (Run)	− 45.5	0.002	9.1	0.375	− 57.7	0.002
0.3 (Walk)	0.8 (Run)	− 48.3	0.002	13.1	0.275	− 61.1	0.002
0.3 (Walk)	1.0 (Run)	− 50.0	0.002	7.4	0.322	− 64.4	0.002
0.6 (Run)	0.8 (Run)	− 5.2	0.020	3.6	0.770	− 8.1	0.027
0.6 (Run)	1.0 (Run)	− 8.2	0.004	− 1.5	1.000	− 15.8	0.010
0.8 (Run)	1.0 (Run)	− 3.2	0.322	− 5.0	0.492	− 8.4	0.027

% diff calculated as ((Froude A–Froude B)/Froude B)*100. $\alpha = 0.00\overline{3 }$ for 15 comparisons across both methods. Comp, compression; AP, anteroposterior; ML, mediolateral.

**Table 10:** Means and standard deviations (S.D.) of root mean squares of ensemble averaged lumbosacral (L5/S1) and thoracolumbar (T12/L1) 3D joint reaction forces (normalized to body weight) by method (inertial measurement unit [IMU] or optical motion capture [OMC]) across conditions.

Method	L5/S1			T12/L1		
	Comp	AP shear	ML shear	Comp	AP shear	ML shear
	Mean (S.D.)	Mean (S.D.)	Mean (S.D.)	Mean (S.D.)	Mean (S.D.)	Mean (S.D.)
IMU	1.735 (0.702)	0.442 (0.186)	0.046 (0.027)	1.138 (0.451)	0.099 (0.025)	0.060 (0.032)
OMC	1.754 (0.759)	0.642 (0.306)	0.096 (0.093)	1.040 (0.387)	0.114 (0.045)	0.064 (0.038)

Comp, compression; AP, anteroposterior; ML, mediolateral.

**Table 11:** Nonparametric Friedman’s tests and percent differences regarding the effect of method (inertial measurement unit [IMU] or optical motion capture [OMC]) on root mean squares of lumbosacral (L5/S1) and thoracolumbar (T12/L1) joint reaction forces across conditions.

Joint	Comp		AP shear		ML shear	
	% diff	*P*	% diff	*P*	% diff	*P*
L5/S1	− 1.1	0.842	− 31.1	< 0.001	− 52.6	0.001
T12/L1	9.5	0.046	− 13.1	0.317	− 5.6	1.000

% diff calculated as ((IMU – OMC)/OMC)*100. $\alpha = 0.05$. Comp, compression; AP, anteroposterior; ML,mediolateral.

Post hoc tests found that L5/S1 and T12/L1 *RMS* 3D joint moment and all other reaction force magnitudes during running conditions were significantly higher than during walking conditions (Tables 5 and 9). For some axis-joint pairs, post hoc tests also found that *RMS* joint moments and reaction forces were higher for faster conditions within walking and/or running gaits (Tables 5 and 9). Friedman’s tests revealed that, relative to OMC models, IMU models overestimated L5/S1 and T12/L1 FE moments by 42.2% and 29.9%, respectively. IMU models also overestimated L5/S1 LB moments by 42.7%, underestimated T12/L1 LB moments by 25.6%, overestimated L5/S1 AR moments by 13.2%, overestimated T12/L1 compression forces by 9.5%, and underestimated L5/S1 AP and ML shear forces by 31.1% and 52.6% (Tables 7 and 11). Overall, relative to OMC, IMU overestimated joint moments by 20.9% and underestimated joint reaction forces by 15.7%.

## Discussion

This study compared IMU and OMC estimates of L5/S1 and T12/L1 3D joint angles, moments, and reaction forces across six speeds, including walking and running. We hypothesized that IMU estimates would not differ significantly from OMC estimates, which are generally considered the best available method for measuring kinematics. In general, this hypothesis was supported. With a few exceptions which we discuss below, comparisons between IMU and OMC dependent variables have low *NRMSE* and high, positive *R* values, indicating the signals are similar in magnitude and trend (Tables 1, 2, 3, Figs. 2, 3, 4, 5, 6, 7).

We also tested the effect of condition and method on estimates of L5/S1 and T12/L1 3D joint moment and reaction force magnitudes. As expected, *RMS* values of L5/S1 and T12/L1 3D joint moments and reaction forces during running were generally higher than during walking (Tables 5 and 9; Figs. 8, 9, 10, 11, 12, 13). Across conditions, *RMS* values of FE and AR moments were higher for IMU relative to OMC, while *RMS* values of L5/S1 and T12/L1 LB moments were higher and lower for IMU relative to OMC, respectively (Table 7, Figs. 8, 9, 10). Across all conditions, *RMS* values for L5/S1 compression forces did not differ between methods, but for T12/L1, IMU values were higher relative to OMC values (Table 11, Fig. 11). *RMS* values for IMU L5/S1 AP and ML shear forces were lower relative to OMC values, with no differences between methods at the T12/L1 joint (Table 11, Figs. 12, 13). These results suggest MSK models that use IMU kinematics may overestimate L5/S1 and T12/L1 FE moments, underestimate T12/L1 LB moments, overestimate T12/L1 compression forces, and underestimate L5/S1 AP and ML shear forces. These considerations would not seem to impact within-method comparisons of spine kinetics.

The highest *NRMSE* values we report are for AP and ML shear forces (Table 3). This is likely due to OMC AP shear forces having 31.1% higher magnitudes than those estimated from IMUs (Table 11). One possible explanation for this is that across conditions, the average degree of sagittal pelvic tilt was ~ 3 deg more flexed for OMC models relative to IMU models. Similarly, OMC ML shear forces were 52.6% higher in magnitude than those estimated from IMUs (Table 11). Note also that average degree of coronal pelvic list was ~ 2 deg more flexed for OMC models relative to IMU models. However, ML shear forces were considerably smaller in magnitude than AP shear and compression forces (Table 8), so offsets between IMU and OMC values can be assumed negligible for walking and running.

A few low-to-medium effect cross-correlations (< 0.3) between IMU and OMC joint angles for five axis-condition pairs (14% of comparisons, see Table 1) appear to be caused by additional frequency components in the OMC signal or a relatively higher OMC signal range. A post hoc Fourier analysis of the OMC signal showed additional frequency components below 4 Hz for the T12/L1 LB angles during running at a Fr of 0.8 and a Fr of 1.0 and the T12/L1 AR angle during running at a Fr of 1.0. These components were absent from the IMU signal and caused *R* values for these comparisons to be lower because the OMC signal reversed its trend relative to the IMU signal twice during a stride. The OMC-estimated L5/S1 LB angle ranges during running at a Fr of 0.6 and 1.0 were 193% and 833% higher than the IMU L5/S1 LB angle ranges, which may explain the lower *R* values calculated between these signals.

Another possible explanation for lower cross-correlations and *NRMSE* values is that for five axis-condition joint angle pairs (14% of comparisons, see Table 1), and also three axis-condition joint reaction force pairs (8% of comparisons, see Table 3), lags were $\ge$ 25% of the stride cycle. This may be due to differences in how we calculated the kinematics files for each method. For OMC, we used the OpenSim Inverse Kinematics Tool, whereas for IMU we converted spine segment angle outputs and pelvis IMU accelerations from Noraxon MR3.18 software into OpenSim-compatible inverse kinematics motion files. This involved multiplying the spine segment angles by the proportion of angular motion attributed to each intervertebral joint according to the Thoracolumbar Spine and Ribcage model six degree of freedom constraints. It also involved applying a low-pass filter, double integrating, and then applying a high-pass filter to the pelvis IMU acceleration signal to estimate pelvis positions in the OpenSim world frame (see Methods). Further, IMU sensors and OMC reflective markers had to be affixed to different locations on the spine because of spatial constraints.

Another possible explanation for the lags regards scaling. We adjusted the marker positions of OMC models; being marker-less, we could not do this for IMU models. As a result, there were slight differences in the inertia and centers of mass of the individual bodies of the IMU and OMC models. These different approaches to calculating the inverse kinematics motion files and scaling may have introduced some variation and error in the signals that apparently caused % stride cycle lags to be higher for a small number of joint angle and reaction force signal pairs.

This is the first study to compare IMU and OMC estimates of L5/S1 and T12/L1 3D joint angles, moments and reaction forces during both walking and running. Because comparisons were done using data pooled across five participants, values of dependent variables include inter-participant variation and are thus more conservative. In addition, a few studies have combined IMU kinematics of the upper body with MSK modeling and this is the first to use the OpenSim Thoracolumbar Spine and Ribcage model. Our results are comparable to the only other IMU validation study of spine kinetics during gait, which compared IMU and OMC estimates of lumbar and thoracic joint moments and forces during walking and reported *NRMSE* values of 0.06–0.17, within the range of values in this study^30^. Other IMU validation studies of spine kinetics have not investigated walking nor running and report only dimensionalized errors, making it difficult to compare their results with ours^28^.

L5/S1 and T12/L1 3D joint moment and reaction force magnitudes during walking in this study are comparable to those estimated with OMC systems reported in the literature. An EMG-optimization model of the lower back estimated maximum L4/L5 moments in FE, LB, and AR of 1.8%, 2.6%, and 0.7% of body weight times height, respectively^5^. These values are similar to our results except for the LB moment. Other link-segment and MSK models of the lower back during walking estimated compression forces of 1.0–2.5 times body weight and peak AP shear forces of ~ 0.6 times body weight, which are comparable to the force values during walking in this study^5,11,52–57^. L5/S1 moments at heel strike during running at 3.8 m s^-1^ were previously estimated to be 2.1, 0.1, and 0.6 N kg^-1^ in FE, LB, and AR, respectively^7^. When made non-dimensional using the reported mean height of participants, these L5/S1 moments are higher than our results, which is unsurprising given their higher running speed and their use of a bottom-up inverse dynamics modeling approach that is more sensitive to heel strike impulses^7^.

Overall, our results support the validity of IMU MSK models for estimating spine kinematics and kinetics. IMU technology opens the possibility of measuring spine movement and tissue loading in nonindustrial environments where OMC technology is often impractical due to lack of necessary infrastructure and inconsistent access to electricity. To better understand links between PA and spine tissue loading, it is necessary to compare age-matched groups with varying PA levels and subsistence patterns, such as hunter-gatherers, horticulturalists, and farmers^44^. This would allow researchers to more rigorously test the hypothesis that LBP is a mismatch condition partly due to the human body being poorly and/or inadequately adapted to persistently reduced levels of PA^41–43,58^.

In addition to studying spine tissue loading with nonindustrial populations, future work should also test how variation in PA and spine tissue loading are associated with factors known to be linked with LBP, including trunk muscle fatigue resistance and strength, vertebral strength and fracture risk, hyperkyphosis, and intervertebral disc narrowing^3,19,59,60^. Our results suggest that combining IMU technology with MSK modeling is a useful method for accurately measuring spine kinematics and kinetics.

### Limitations

There are several limitations to this study. The sample size was small (*n* = 5), albeit comparable to other studies of spine tissue loading during gait and equal to the sample size of the only other IMU validation study that involved walking^5,11,30^. Participants walked and ran on a double belt, force plate-instrumented treadmill, and differences have been reported for spine kinematics and GRFs for treadmill and overground walking^61^. We did not use full-body OMC and IMU setups, although lower limb contributions to lower back loads are minimal compared to the upper body; most low back loading results from stabilizing the inertial mass of the upper body^62,63^. We increased the number of OMC markers from 24 to 27 for the last three participants, which may have led to slight differences in joint angle, moment, and reaction force estimates between participants. The study was relatively short in duration, as each experimental condition lasted 60 s. Future work should test the validity of IMU estimates of spine tissue loading over longer durations to assess potential effects of signal drift^27^. The OpenSim models used a top-down modeling approach to facilitate kinetic comparisons with the IMU models (which didn’t incorporate GRFs), and to make the findings applicable to field research where GRFs will often be unavailable. Studies have shown low back loading during gait to be sensitive to top-down vs. bottom-up modeling approaches because bottom-up approaches are more sensitive to heel strike impulses^5,6,64^. Future work should compare top-down and bottom-up approaches with this OpenSim model. The OpenSim model did not include passive force contributions (i.e., from muscles, ligaments, intervertebral discs), but since this was the case for both IMU and OMC, this likely did not impact any differences between methods. We did not incorporate electromyography (EMG) optimization into our modeling approach. L5/S1 3D joint reaction forces have been shown to be sensitive to recorded trunk muscle EMG, however the magnitude of these differences is low ($\pm$ 4%) and would most likely not impact comparisons between our tasks^65^.

## Conclusion

We found that IMU estimates of L5/S1 and T12/L1 3D joint angles, moments, and reaction forces during walking and running were generally similar in magnitude and trend to values estimated using OMC. As expected, L5/S1 and T12/L1 3D joint moments and reaction forces were higher during running than walking. Relative to OMC, IMU-created MSK models appear to overestimate joint moments and underestimate joint reaction forces. Our results suggest that using IMU technology combined with MSK spine modeling is a valid means for measuring spine movement and tissue loading that could be applied in non-laboratory, real-world contexts, including nonindustrial environments. Because people living in nonindustrial environments typically have relatively higher PA levels than their industrialized counterparts, they may be invaluable for testing hypotheses about the effects of reduced and unnatural spine movement and tissue loading on LBP^41^.

### Supplementary Information

Supplementary Tables.

## Data Availability

The datasets generated and/or analyzed during the current study are available from the corresponding author on reasonable request.

## References

[1] Hartvigsen J (2018). What low back pain is and why we need to pay attention. The Lancet.

[2] Hoy D (2012). A systematic review of the global prevalence of low back pain. Arthritis Rheum..

[3] Adams MA, Bogduk N, Burton K, Dolan P (2006). The Biomechanics of Back Pain.

[4] Foster NE (2018). Prevention and treatment of low back pain: Evidence, challenges, and promising directions. The Lancet.

[5] Callaghan JP, Patla AE, McGill SM (1999). Low back three-dimensional joint forces, kinematics, and kinetics during walking. Clin. Biomech..

[6] Hendershot BD, Wolf EJ (2014). Three-dimensional joint reaction forces and moments at the low back during over-ground walking in persons with unilateral lower-extremity amputation. Clin. Biomech..

[7] Seay J, Selbie WS, Hamill J (2008). In vivo lumbo-sacral forces and moments during constant speed running at different stride lengths. J. Sports Sci..

[8] Banks JJ (2023). Using static postures to estimate spinal loading during dynamic lifts with participant-specific thoracolumbar musculoskeletal models. Appl. Ergon..

[9] Raabe ME, Chaudhari AMW (2016). An investigation of jogging biomechanics using the full-body lumbar spine model: Model development and validation. J. Biomech..

[10] Actis JA (2018). Validation of lumbar spine loading from a musculoskeletal model including the lower limbs and lumbar spine. J. Biomech..

[11] Arshad R (2018). Spinal loads and trunk muscles forces during level walking—A combined in vivo and in silico study on six subjects. J. Biomech..

[12] Bruno AG, Bouxsein ML, Anderson DE (2015). Development and validation of a musculoskeletal model of the fully articulated thoracolumbar spine and rib cage. J. Biomech. Eng..

[13] Senteler M, Weisse B, Rothenfluh DA, Snedeker JG (2016). Intervertebral reaction force prediction using an enhanced assembly of OpenSim models. Comput. Methods Biomech. Biomed. Eng..

[14] Meng X (2015). Incorporating six degree-of-freedom intervertebral joint stiffness in a lumbar spine musculoskeletal model—Method and performance in flexed postures. J. Biomech. Eng..

[15] Beaucage-Gauvreau E (2019). Validation of an OpenSim full-body model with detailed lumbar spine for estimating lower lumbar spine loads during symmetric and asymmetric lifting tasks. Comput. Methods Biomech. Biomed. Eng..

[16] Christophy M, Faruk Senan NA, Lotz JC, O’Reilly OMA (2012). Musculoskeletal model for the lumbar spine. Biomech. Model Mechanobiol..

[17] Delp SL (2007). OpenSim: Open-source software to create and analyze dynamic simulations of movement. IEEE Trans. Biomed. Eng..

[18] Bruno AG, Anderson DE, D’Agostino J, Bouxsein ML (2012). The effect of thoracic kyphosis and sagittal plane alignment on vertebral compressive loading. J. Bone Min. Res..

[19] Bruno AG, Burkhart K, Allaire B, Anderson DE, Bouxsein ML (2017). Spinal loading patterns from biomechanical modeling explain the high incidence of vertebral fractures in the thoracolumbar region. J. Bone Min. Res..

[20] Burkhart KA, Bruno AG, Bouxsein ML, Bean JF, Anderson DE (2018). Estimating apparent maximum muscle stress of trunk extensor muscles in older adults using subject-specific musculoskeletal models. J. Orthop. Res..

[21] Bruno AG (2017). Incorporation of CT-based measurements of trunk anatomy into subject-specific musculoskeletal models of the spine influences vertebral loading predictions. J. Orthop. Res..

[22] Burkhart K, Grindle D, Bouxsein ML, Anderson DE (2020). Between-session reliability of subject-specific musculoskeletal models of the spine derived from optoelectronic motion capture data. J. Biomech..

[23] O’Grady M (2021). Measuring spinal mobility using an inertial measurement unit system: a reliability study in axial spondyloarthritis. Diagnostics (Basel).

[24] Weygers I (2020). Inertial sensor-based lower limb joint kinematics: A methodological systematic review. Sensors.

[25] van der Kruk E, Reijne MM (2018). Accuracy of human motion capture systems for sport applications: State-of-the-art review. Eur. J. Sport Sci..

[26] Robert-Lachaine X, Mecheri H, Larue C, Plamondon A (2017). Validation of inertial measurement units with an optoelectronic system for whole-body motion analysis. Med. Biol. Eng. Comput..

[27] Al Borno M (2022). OpenSense: An open-source toolbox for inertial-measurement-unit-based measurement of lower extremity kinematics over long durations. J. Neuroeng. Rehabil..

[28] Lee CJ, Lee JK (2022). Inertial motion capture-based wearable systems for estimation of joint kinetics: A systematic review. Sensors.

[29] Muller A, Mecheri H, Corbeil P, Plamondon A, Robert-Lachaine X (2022). Inertial motion capture-based estimation of L5/S1 moments during manual materials handling. Sensors.

[30] Khurelbaatar T, Kim K, Lee S, Kim YH (2015). Consistent accuracy in whole-body joint kinetics during gait using wearable inertial motion sensors and in-shoe pressure sensors. Gait Posture.

[31] Mannion AF, Müntener M, Taimela S, Dvorak J (2001). Comparison of three active therapies for chronic low back pain: Results of a randomized clinical trial with 1-year follow-up. Rheumatology.

[32] McGill S (2015). Back Mechanic.

[33] Lahad A (1994). The effectiveness of four interventions for the prevention of low back pain. JAMA: J. Am. Med. Assoc..

[34] Biering-Sørensen F (1984). Physical measurements as risk indicators for low-back trouble over a one-year period. Spine.

[35] Alaranta H, Luoto S, Heliövaara M, Hurri H (1995). Static back endurance and the risk of low-back pain. Clin. Biomech..

[36] Sibson BE (2021). Trunk muscle endurance, strength and flexibility in rural subsistence farmers and urban industrialized adults in western Kenya. Am. J. Hum. Biol..

[37] Castillo ER (2016). Physical fitness differences between rural and urban children from western Kenya. Am. J. Hum. Biol..

[38] Lieberman DE (2020). Exercised: Why Something We Never Evolved to do is Healthy and Rewarding.

[39] Raichlen DA, Lieberman DE (2022). The evolution of human step counts and its association with the risk of chronic disease. Current Biol..

[40] Althoff T (2017). Large-scale physical activity data reveal worldwide activity inequality. Nature.

[41] Castillo, E. R. & Lieberman, D. E. Lower back pain. In *Evolution, Medicine and Public Health,* vol. 56, 2–3. 10.1093/emph/eou034 (2015). 10.1093/emph/eou034 PMC4315061 25577608

[42] Lieberman DE (2013). The Story of the Human Body.

[43] Volinn E (1997). The epidemiology of low back pain in the rest of the world. Spine.

[44] Kraft TS (2021). The energetics of uniquely human subsistence strategies. Science (1979).

[45] Hendershot BD, Shojaei I, Acasio JC, Dearth CL, Bazrgari B (2018). Walking speed differentially alters spinal loads in persons with traumatic lower limb amputation. J. Biomech..

[46] Alexander RM, Jayes AS (1983). A dynamic similarity hypothesis for the gaits of quadrupedal mammals. J. Zool..

[47] Anderson DE, D’Agostino JM, Bruno AG, Manoharan RK, Bouxsein ML (2012). Regressions for estimating muscle parameters in the thoracic and lumbar trunk for use in musculoskeletal modeling. J. Biomech..

[48] Alemi MM (2021). The influence of kinematic constraints on model performance during inverse kinematics analysis of the thoracolumbar spine. Front. Bioeng. Biotechnol..

[49] Winter DA (2009). Biomechancs and Motor Control of Human Movement.

[50] Cohen J (1988). Statistical Power Analysis for the Behavioral Sciences.

[51] Field A, Miles J, Field Z (2012). Discovering Statistics Using R.

[52] Cappozzo A (1983). Compressive loads in the lumbar vertebral column during normal level walking. J. Orthop. Res..

[53] Cappozzo A (1983). The forces and couples in the human trunk during level walking. J. Biomech..

[54] Shojaei I, Hendershot BD, Wolf EJ, Bazrgari B (2016). Persons with unilateral transfemoral amputation experience larger spinal loads during level-ground walking compared to able-bodied individuals. Clin. Biomech..

[55] Khoo BCC, Goh JCH, Bose K (1995). A biomechanical model to determine lumbosacral loads during single stance phase in normal gait. Med. Eng. Phys..

[56] Cromwell R, Schultz AB, Beck R, Warwick D (1989). Loads on the lumbar trunk during level walking. J. Orthop. Res..

[57] Banks JJ, Umberger BR, Boyer KA, Caldwell GE (2022). Lower back kinetic demands during induced lower limb gait asymmetries. Gait Posture.

[58] Hoy D, Toole MJ, Morgan D, Morgan C (2003). Low back pain in rural Tibet. The Lancet.

[59] Stieglitz J (2019). Computed tomography shows high fracture prevalence among physically active forager-horticulturalists with high fertility. Elife.

[60] McGill S (2007). Low Back Disorders: Evidence-Based Prevention and Rehabilitation.

[61] Riley PO, Paolini G, Della Croce U, Paylo KW, Kerrigan DC (2007). A kinematic and kinetic comparison of overground and treadmill walking in healthy subjects. Gait Posture.

[62] Winter D (1995). Human balance and posture control during standing and walking. Gait Posture.

[63] Castillo ER, Hsu C, Mair RW, Lieberman DE (2017). Testing biomechanical models of human lumbar lordosis variability. Am. J. Phys. Anthropol..

[64] Kingma I, de Looze MP, Toussaint HM, Klijnsma HG, Bruijnen TBM (1996). Validation of a full body 3-D dynamic linked segment model. Hum. Mov. Sci..

[65] Banks JJ, Umberger BR, Caldwell GE (2022). EMG optimization in OpenSim: A model for estimating lower back kinetics in gait. Med. Eng. Phys..

