# Supplementary material for: Using inertial measurement units to estimate spine joint kinematics and kinetics during walking and running
Source: Sci Rep. 2024 Jan 2;14:234. doi: 10.1038/s41598-023-50652-w (PMC10762015; doi:10.1038/s41598-023-50652-w)
Supplement: Supplementary file 1 — Supplementary Tables. [file 41598_2023_50652_MOESM1_ESM.pdf]

Supplemental Information (SI) for Using inertial measurement units to estimate spine joint kinematics and kinetics during walking and running

Authors

Benjamin E. Sibson [1]*
Jacob J. Banks [2,3]
Ali Yawar [1]
Andrew K. Yegian [1]
Dennis E. Anderson [2,3]
Daniel E. Lieberman [1]

Affiliations

[1] Department of Human Evolutionary Biology, Harvard University, Cambridge, MA, USA
[2] Center for Advanced Orthopedic Studies, Beth Israel Deaconess Medical Center, Boston, MA, USA
[3] Department of Orthopedic Surgery, Harvard Medical School, Boston, MA, USA

*Corresponding author email: bsibson@g.harvard.edu

1. SI Tables 1 – 4

SI Table 1. Summary statistics for Friedman's tests of the effect of Froude number (Fr) and method (inertial measurement unit vs. optical motion capture) on dimensionless lumbosacral joint moment *RMS* values. FE = flexion-extension; LB = lateral bending; AR = axial rotation; SS = sum of squares; df = degrees of freedom; MS = mean squares; χ^2= chi-squared test statistic. $\alpha = 0.05$.

**Axis**	**Model Factor**	**SS**	**df**	**MS**	χ^2	***P***
FE	Condition (Fr)	3302.6	5	660.5	42.6	<0.001
	Interaction	22.8	5	4.6		
	Error	1169.6	48	24.4		
	Total	4495.0	59			
	Method	11.3	1	11.3	6.8	0.009
	Interaction	9.7	14	0.7		
	Error	54.0	30	1.8		
	Total	75.0	59			
LB	Condition (Fr)	3551.8	5	710.4	45.8	<0.001
	Interaction	24.0	5	4.8		
	Error	919.2	48	19.2		
	Total	4495.0	59			
	Method	24.1	1	24.1	14.4	<0.001
	Interaction	12.9	14	0.9		
	Error	38.0	30	1.3		
	Total	75.0	59			
AR	Condition (Fr)	3648.4	5	729.7	47.1	<0.001
	Interaction	11.0	5	2.2		
	Error	835.6	48	17.4		
	Total	4495.0	59			
	Method	17.1	1	17.1	10.2	0.001
	Interaction	8.9	14	0.6		
	Error	49.0	30	1.6		
	Total	75.0	59			

SI Table 2. Summary statistics of Friedman's tests of the effect of Froude number (Fr) and method (inertial measurement unit vs. optical motion capture) on dimensionless thoracolumbar joint moment *RMS* values. FE = flexion-extension; LB = lateral bending; AR = axial rotation; SS = sum of squares; df = degrees of freedom; MS = mean squares; χ^2= chi-squared test statistic. $\alpha = 0.05$.

Axis	Model Factor	SS	df	MS	χ^2	*P*
FE	Condition (Fr)	3387.8	5	677.6	43.7	<0.001
	Interaction	1.6	5	0.3		
	Error	1105.6	48	23.0		
	Total	4495.0	59			
	Method	6.7	1	6.7	4.0	0.046
	Interaction	9.3	14	0.7		
	Error	59.0	30	2.0		
	Total	75.0	59			
LB	Condition (Fr)	3588.8	5	717.1	46.3	<0.001
	Interaction	74.6	5	14.9		
	Error	831.6	48	17.3		
	Total	4495.0	59			
	Method	17.1	1	17.1	10.2	0.001
	Interaction	12.9	14	0.9		
	Error	45.0	30	1.5		
	Total	75.0	59			
AR	Condition (Fr)	3745.4	5	749.1	48.3	<0.001
	Interaction	2.0	5	0.4		
	Error	747.6	48	15.6		
	Total	4495.0	59			
	Method	3.3	1	3.3	2.0	0.162
	Interaction	23.7	14	1.7		
	Error	48.0	30	1.6		
	Total	75.0	59			

SI Table 3. Summary statistics for Friedman's tests of the effect of Froude number (Fr) and method (inertial measurement unit vs. optical motion capture) on dimensionless lumbosacral joint force *RMS* values. AP = anteroposterior; ML = mediolateral; SS = sum of squares; df = degrees of freedom; MS = mean squares; χ^2= chi-squared test statistic. $\alpha = 0.05$.

**Axis**	**Model Factor**	**SS**	**df**	**MS**	χ^2	***P***
Compression	Condition (Fr)	3606.4	5	721.3	46.5	<0.001
	Interaction	13.0	5	2.6		
	Error	875.6	48	18.2		
	Total	4495.0	59			
	Method	0.1	1	0.1	0.0	0.842
	Interaction	14.9	14	1.1		
	Error	60.0	30	2		
	Total	75.0	59			
AP shear	Condition (Fr)	3585.0	5	717.0	46.3	<0.001
	Interaction	18.8	5	3.8		
	Error	891.2	48	18.6		
	Total	4495.0	59			
	Method	48.6	1	48.6	29.2	<0.001
	Interaction	2.4	14	0.2		
	Error	24.0	30	0.8		
	Total	75.0	59			
ML shear	Condition (Fr)	2219.4	5	443.9	28.6	<0.001
	Interaction	198.8	5	39.8		
	Error	2076.8	48	43.3		
	Total	2295.0	59			
	Method	17.1	1	17.1	10.2	0.001
	Interaction	22.9	14	1.6		
	Error	35.0	30	1.2		
	Total	75.0	59			

SI Table 4. Summary statistics for Friedman's tests of the effect of Froude number (Fr) and method (inertial measurement unit vs. optical motion capture) on dimensionless thoracolumbar joint force *RMS* values. AP = anteroposterior; ML = mediolateral; SS = sum of squares; df = degrees of freedom; MS = mean squares; χ^2= chi-squared test statistic. $\alpha = 0.05$.

Axis	Model Factor	SS	df	MS	χ^2	*P*
Compression	Condition (Fr)	3617.0	5	723.4	46.7	<0.001
	Interaction	12.4	5	2.5		
	Error	865.6	48	18.0		
	Total	4495.0	59			
	Method	6.7	1	6.7	4.0	0.046
	Interaction	11.3	14	0.8		
	Error	57.0	30	1.9		
	Total	75.0	59			
AP shear	Condition (Fr)	833.0	5	166.6	10.8	0.056
	Interaction	27.6	5	5.5		
	Error	3634.4	48	75.7		
	Total	4495.0	59			
	Method	1.7	1	1.7	1.0	0.317
	Interaction	17.3	14	1.2		
	Error	56.0	30	1.9		
	Total	75.0	59			
ML shear	Condition (Fr)	3632.0	5	726.4	46.9	<0.001
	Interaction	47.0	5	9.4		
	Error	816.0	48	17.0		
	Total	4495.0	59			
	Method	0.0	1	0.0	0.0	1.000
	Interaction	16.0	14	1.1		
	Error	59.0	30	2.0		
	Total	75.0	59			
